# Interaction of Doxorubicin Embedded into Phospholipid Nanoparticles and Targeted Peptide-Modified Phospholipid Nanoparticles with DNA

**DOI:** 10.3390/molecules28145317

**Published:** 2023-07-10

**Authors:** Veronica V. Pronina, Lyubov V. Kostryukova, Tatiana V. Bulko, Victoria V. Shumyantseva

**Affiliations:** 1 Institute of Biomedical Chemistry, Pogodinskaya Street, 10, Build 8, 119121 Moscow, Russia; veronicapunch@mail.ru (V.V.P.); kostryukova87@gmail.com (L.V.K.); tanya.bulko@mail.ru (T.V.B.); 2 Faculty of Biochemistry, Pirogov Russian National Research Medical University, Ostrovitianov Street, 1, 117997 Moscow, Russia

**Keywords:** doxorubicin, DNA, drug–DNA interaction, electrochemical monitoring, nanoliposomes, NGR peptide

## Abstract

The interactions of dsDNA with new targeted drug delivery derivatives of doxorubicin (DOX), such as DOX embedded into phospholipid nanoparticles (NPhs) and DOX with the NGR targeted peptide-modified NPhs were studied electrochemically by differential pulse voltammetry technique. Screen-printed electrodes (SPEs), modified with stable fine dispersions of carbon nanotubes (CNTs), were used for quantitative electrochemical investigations of direct electrochemical oxidation of guanine, adenine, and thymine heterocyclic bases of dsDNA, and their changes in the presence of DOX nanoderivatives. Analysing the shifts of peak potentials of nucleobases in the presence of drug, we have shown that the doxorubicin with NGR targeted peptide changed the mode of interaction in DNA–drug complexes from intercalative to electrostatic. Binding constants (*K_b_*) of DNA–drug complexes were calculated in accordance with adenine, guanine, and thymine oxidation signals. Based on our experiments, we have proven that the surface modification of a drug delivery system with NGR targeted peptide dramatically changed the mechanism of interaction of drug with genetic material. DNA-mediated drug toxicity was calculated based on the concentration-dependent “response” of heterocyclic nucleobases on drug influence. DOX, DOX-loaded phospholipid nanoparticles (NPhs), and DOX with NGR addressed peptide-modified NPhs were moderately toxic in the concentration range of 0.5–290 µM.

## 1. Introduction

**Scheme 1:**
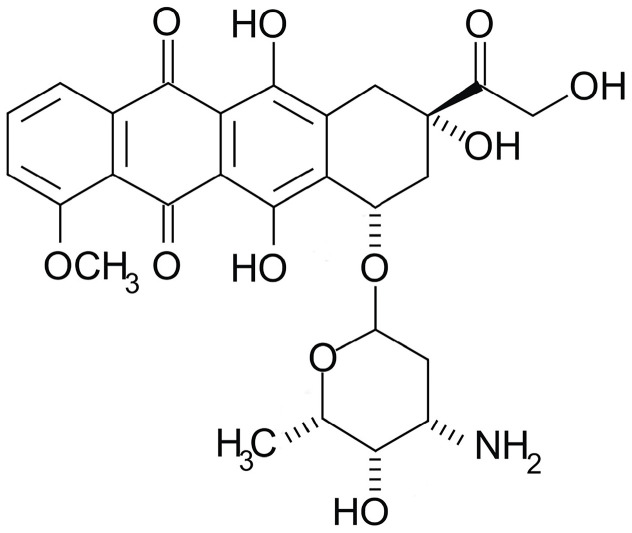
Chemical structure of doxorubicin.

Doxorubicin (DOX) is an anthracycline-based antitumor and antibiotic drug derived from the bacterium *Streptomyces peucetius*, which is part of the anthracycline chemotherapy drug group. Structurally, DOX possesses an amino sugar and four rings typical of an anthraquinone structure (Scheme 1). DOX, a widely used cancer drug, is applied to treat breast, lung, stomach, ovarian, thyroid cancer, soft tissue and bone sarcomas, gastric, non-Hodgkin’s and Hodgkin’s lymphoma, sarcoma, pediatric cancers, leukemia, and multiple myeloma [1]. Mechanisms of DOX toxicity have been described previously [2,3,4,5,6]. The main mechanism of pharmacological activity of doxorubicin involves the ability of the drug to intercalate into DNA base pairs, and/or covalently bind to proteins involved in DNA replication and transcription, leading to inhibition of both DNA and RNA synthesis. DOX inhibits the enzyme topoisomerase II, which is explained by DOX intercalation into DNA, causing DNA damage and induction of apoptosis [2,3]. DOX oxidation to semiquinone, an unstable metabolite, which is converted back to DOX in a process, releases reactive oxygen species that cause DNA damage and lipid peroxidation [4,5,6]. Because of this process, reactive oxygen species are redistributed in the cell, which can lead to more intensive lipid peroxidation and membrane damage, DNA damage, oxidative stress, and triggering of apoptotic pathways of cell death [2,3,4,5,6].

**Scheme 2:**
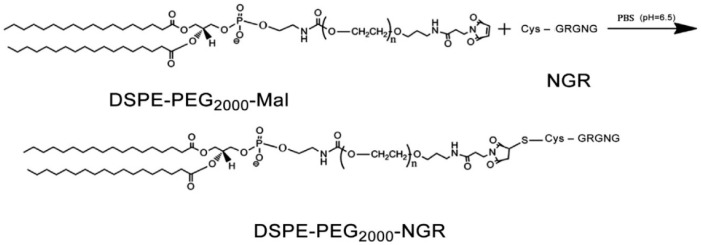
Synthesis of DOX embedded in phospholipid nanoparticles (NPhs) with the targeted peptide NGR.

However, DOX does not selectively interact with its targets and is randomly distributed in the body, resulting in toxic and adverse effects, provoking such pathologic processes as myelosuppression, nephrotoxicity, dose-dependent cardiotoxicity, and multidrug resistance. In particular, the manifestation of severe cardiotoxicity upon accumulation can cause myocardial damage, and even heart failure. Thus, the clinical use of DOX is limited to some extent due to serious toxic adverse effects [3,7]. One approach to overcome these shortcomings is the development of DOX-loaded liposomes and targeted delivery systems. Liposomes, as nanocarriers, are the most successful delivery platform in terms of safety and efficacy [8]. Their applicability spans small molecule therapies, gene therapy, immunotherapy, and diagnostic and theranostic applications. Drug encapsulation into liposomes, without the inclusion of additional agents, is a passive transport of drugs, and its use is limited in the clinic, in particular the inclusion of DOX in liposomes. However, liposomes are, in their own way, an effective platform to which various guiding fragments can be attached. Modification of liposomes with specific ligands can enhance their targeting efficiency. Various compounds are used as such systems: aptamers [9], antibodies [10], vitamins [11,12], and peptides [13,14,15,16]. Recently, increasing interest has been paid to targeted peptides as guiding fragments [17,18,19,20,21,22,23] for cancer-special receptors. Aminopeptidase N (APN/CD13) is a multifunctional glycoprotein with peptidase, receptor, and signaling roles in cancer tissues. APN activity has been associated with the progression of many types of cancer, indicating significant therapeutic potential for the treatment of cancer. The NGR peptide (with the asparagine-glycine-arginine amino acid sequence) targets the APN isoform expressed on endothelial cells in angiogenic vessels, such as new vessels in tumors. The high ligand specificity makes NGR a promising candidate for the active leading of medications to the receptor, such as APN/CD13 [22]. The composition of DOX embedded in phospholipid nanoparticles (NPhs), with and without the targeted peptide NGR, has been developed earlier [23]. The targeted peptide NGR ((NH2-)Gly-Asn-Gly-Arg-Gly-Cys(−COOH)) was attached to soybean phosphatidylcholine Lipoid S100 (DSPE)/polyethylene glycol (PEG) by means of maleimide (Mal) (Scheme 2) [23].

Liposomes and lipid nanocarriers are often employed in drug delivery due to precise targeting of different tissues [8,24]. The developed compositions of DOX, a targeted CD13-directed peptide, have shown their effectiveness in vitro [24].

In our investigation, we performed a comparative study of the mechanism of interaction of DOX, DOX-loaded liposome (as drug-loaded phospholipid nanoparticles NPhs), and DOX with the NGR targeted peptide-modified NPhs with dsDNA. DNA–drug interaction is one of the most important areas of biological research in the development of new drugs, since the binding of molecules to DNA can affect the properties of DNA, leading to its destruction or to a change in the functioning of the genome. DNA is a main biomolecule member in genomics and proteomics. This heteropolymer molecule plays a key role in transcription, translation, replication, and division processes. From another side, DNA is a pharmacological target of drug formulations [2,3,12]. The binding processes of drug molecules to or onto DNA can dramatically change the properties of DNA, leading to disruption in the functioning of the whole genome.

To study the mechanisms of DNA–drug interaction, different physical-chemical methods, such as chromatographic, UV-vis spectral methods (absorption spectroscopy), nuclear magnetic resonance (NMR), FTIR spectroscopic measurements, electrophoretic methods, luminescent and fluorescent methods, atomic force microscopy (AFM), isothermal titration calorimetry, confocal Raman spectroscopy, and mass spectrometry, are widely used [6,25,26]. The investigation of peculiarities and the mode of the medication’s interaction with DNA is an important part of rational drug design construction of new and more efficient therapeutic agents, and prediction of drug influence on genomic processing.

**Scheme 3:**
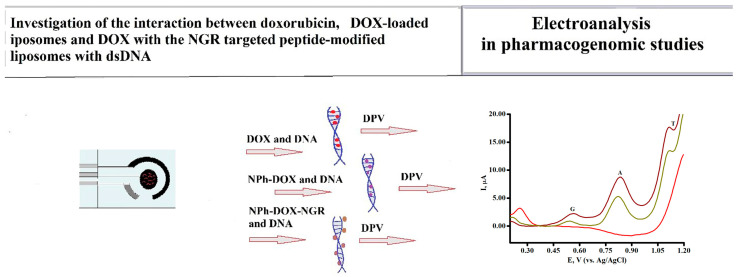
Electrochemical analysis of DOX, DOX embedded in phospholipid nanoparticles (NPhs), and DOX with the targeted peptide NGR interaction with DNA. (**—**) DNA (G, guanine; A, adenine; T, thymine; (**—**) DNA + drug, (-); (**—**) drug.

Electroanalysis in pharmacogenomic studies has proven itself due to high sensitivity of the analysis, low consumption of reagents (2–60 µL), selectivity to biological objects, and nanostructuring of the surface of the working electrode to improve the metrological parameters of the biosensor. Differential pulse voltammetry (DPV) possesses high sensitivity, which permits to register heterocyclic bases separately for the detailed study of drug–DNA interactions (Scheme 3). Intensive research was carried out in recent years to study the mechanisms of complex formation of the DNA–drug, such as, for example, rifampicin and nanosome/rifampicin (rifampicin embedded into lipid nanoparticles) [27], abiraterone acetate [28], and phospholipid nanoparticles with different contents of phosphatidylcholine [29]. There are also extensive studies in this area, in the form of review articles [12,30,31,32], as we have cited. The ability to calculate binding constants and thermodynamic parameters for each of the heterocyclic nucleobases allows registering the effect of the drug on DNA in more detail. The use of disposable screen-printed planar electrodes with a minimum diameter of the working electrode (1–2 mm) makes it possible to miniaturize and standardize the analytical procedures.

The aim of this study is to discover the effects of DOX, or DOX as nanoformulated forms (DOX-loaded NPhs and DOX with the NGR targeted peptide), onto DNA as a model molecule in pharmacogenomics. To assess the mechanism of interaction of medications with DNA, we used electrochemical techniques, which possess high sensitivity, selectivity, and registration of a concentration-dependent “response” of heterocyclic nucleobases on drug influence [12,21,25,26,27,28,29,30,31,32,33,34].

## 2. Results

### 2.1. Electrochemical Behavior of Doxorubicin on SPE/CNT

Doxorubicin (DOX) is an antineoplastic antibiotic with wide pharmacological activity [2,3,7,24,31,34,35,36,37,38]. Analysis of DOX levels is an important task for the determination of this medication in body fluids for the individualization of drug dosage, and for the investigation and evaluation of the mechanism of drug–biomolecule(s) interactions [12,39,40,41].

We studied the electrochemical behavior of DOX on screen-printed electrodes modified with single-wall carbon nanotubes (SPE/CNT). Disposable screen-printed electrodes were used due to their commercial availability, relatively low cost, wide opportunities and range of modification methods, and adaptability for on-site measurements. Nanomaterials, such as carbon nanotubes and metal nanoparticles, significantly improve the sensitivity of electrodes [42,43]. We used a drop-casting method for the modification of SPEs with a dispersion of CNTs in carboxymethylcellulose (SPE/CNT).

**Figure 1:**
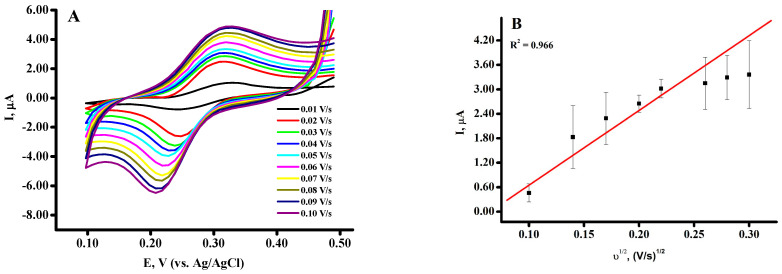
(**A**) Cyclic voltammograms recorded for 100 µM DOX at a scan rate in the range of 0.01–0.10 V/s. (**B**) Dependence of the intensity of electroreduction peak current of doxorubicin on the square root of the scan rate.

Cyclic voltammetry (CV) measurements were employed to study the electrochemical behavior of DOX on the SPE/CNT. Cyclic voltammograms (CV) for DOX in the potential range of +0.1 V–+0.5 V were investigated in electrolyte buffer corresponding to physiological media (0.1 M potassium phosphate buffer with 50 mM NaCl as supporting electrolyte, pH 7.4) (Figure 1). DOX exhibited reversible anodic and cathodic peaks centered at E = +0.269 V (at scan rate of 0.05 V/s). Figure 1A represents the profiles of DOX at scan rate ranges of 0.01–0.1 V/s.

Linear dependence of the intensity of electroreduction/electro-oxidation of doxorubicin on the square root of the scan rate (Figure 1B) proves that the process is a controlled by diffusion.

**Figure 2:**
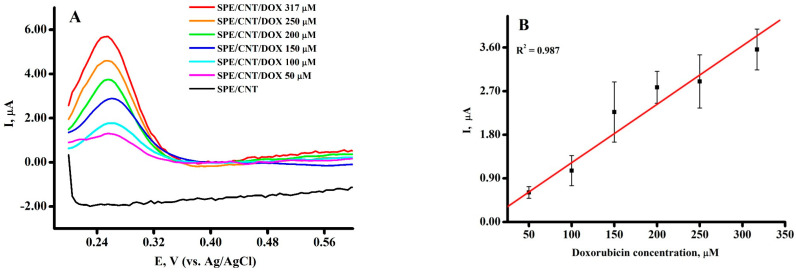
(**A**) Differential pulse voltammetry (DPV) of doxorubicin (DOX) on screen-printed electrodes modified with single-wall carbon nanotubes (SPE/CNT) at a concentration range from 50 to 317 µM. Black line represents DPV of SPE/CNT in supporting electrolyte. (**B**) The plot of the oxidation DPV peak current vs. DOX concentration. The relative standard deviation of the DPV response on the SPE/CNT for three experiments was ±5%.

Differential pulse voltammetry (DPV) for DOX in the potential range of +0.2 ÷ +0.6 V was investigated in electrolyte buffer corresponding to physiological media (0.1 M potassium phosphate buffer with 50 mM NaCl as supporting electrolyte, pH 7.4). DOX exhibited a single anodic peak at E = +0.260 ± 0.006 V (Figure 2A). Electroanalytical parameters of a quantitative DOX assay with SPE/CNT are presented in Table 1. The limit of detection (LOD) and the limit of quantification (LOQ) were calculated from the calibration curve as kSD/b (k = 3 for LOD, k = 10 for LOQ), b = slope of the calibration curve, SD = standard deviation of the intercept [9,44,45].

The proposed electroanalytic approach is a sensitive and robust method for the quantitative determination of DOX.

### 2.2. Investigation of the Interaction between Doxorubicin and DOX-Loaded NPhs with and without the Targeted Peptide NGR with dsDNA

**Figure 3:**
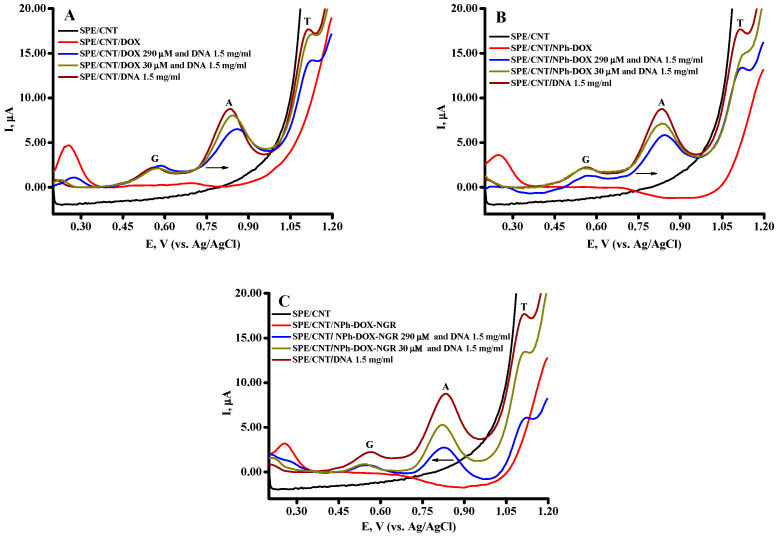
(**A**) Differential pulse voltammetry (DPV) of dsDNA on screen-printed electrodes modified with single-wall carbon nanotubes (SPE/CNT) in the absence (-), in the presence of DOX, SPE/CNT (-), SPE/CNT/DOX (-) 317 µM; (**B**) DPV of dsDNA on SPE/CNT in the absence (-), in the presence of NPh-DOX, SPE/CNT (-), SPE/CNT/NPh-DOX (-), 317 µM; (**C**) DPV of dsDNA on SPE/CNT in the absence (-), in the presence of NPh-DOX-NGR, SPE/CNT (-), SPE/CNT/NPh-DOX-NGR (-), 317 µM. Arrows show the shift of potentials. G is guanine, A is adenine, T is thymine.

**Figure 4:**
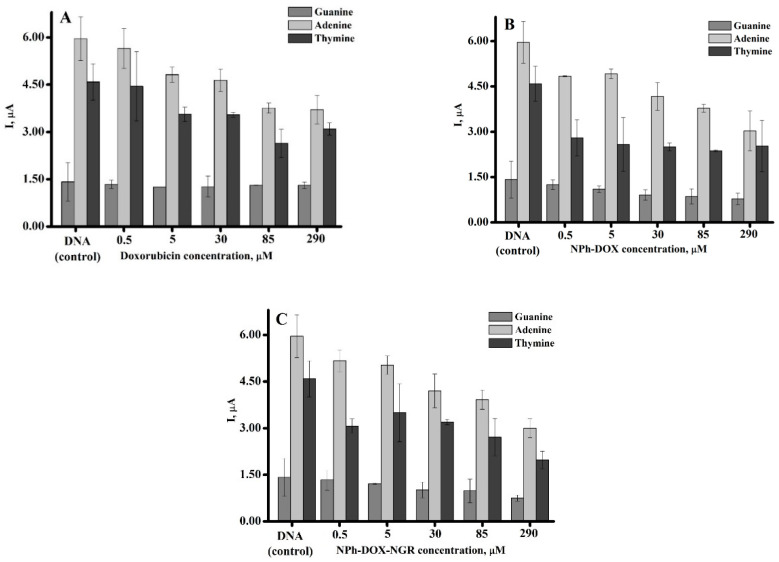
Histograms corresponding to the average guanine, adenine, and thymine signals declined after the addition of DOX (**A**); NPh-DOX (**B**); and NPh-DOX-NGR (**C**) in the range of drug concentration 0.5–290 µM.

The success of therapeutic activity of medicinal preparations depends on the interaction of drug with its target(s). The DNA molecule is one of the main participants of such pharmacological events during medication [9,30,31,46]. The oxidation potential of DOX does not overlap with DNA oxidation signals for heterocyclic bases, registered by means of DPV. This electrochemical behavior of DOX permits to investigate the mechanism of drug–DNA interaction based on registration of the DNA molecule response itself. The drug binding processes were examined by DPV via the registration of a decrease in peak current intensity of guanine, adenine, and thymine of DNA in the presence of doxorubicin, DOX-loaded NPhs (NPh-DOX), or DOX-loaded NPhs with the NGR targeted peptide-modified liposomes (NPh-DOX-NGR). After the interaction of DOX and nanoderivatives with DNA, the concentration-dependent decline of G, A, and T peak currents was observed (Figure 3A–C and Figure 4A–C). It is important to note that both purine bases guanine and adenine, and pyrimidine base thymine are sensitive to the drug influence on oxidation peak current.

Shifts of electro-oxidation peak potentials of heterocyclic bases are well-known characteristics of drug–DNA interactions [12,13,32,34,44,46]. The positive shifts of the peak potential are typical for the intercalative hydrophobic mode of drug binding, which leads to difficulty in the electron transfer processes. The negative shift reflects the binding mode as electrostatic interaction [12,46]. These parameters were analysed for DOX, NPh-DOX, and NPh-DOX-NGR.

It is important to underline the shifts of oxidation potentials of nucleobases G, A, and T in the positive direction as 20–30 mV in the presence of DOX and NPh-DOX (Figure 3A,B), with the most pronounced effect for G and A nucleobases. The standard deviation of potentials did not exceed 5%. The mechanism of DOX interaction with DNA was studied earlier, and it was shown that the intercalative mode into DNA base pairs is predominant [1,2,3,4,5,31] due to the interaction of aromatic moieties of DOX. The anodic shifts of oxidation potentials of G and A registered with DPV technique confirms this intercalative ability of DOX and NPh-DOX interaction mode [32,44,47,48].

In contrast to DOX and NPh-DOX, doxorubicin with a targeted component, peptide with an NGR motif (NPh-DOX-NGR), shifts the oxidation potentials of nucleobases G and A in the negative direction as 8–30 mV (Figure 3C). This phenomenon may reflect the influence of a charged peptide fragment on the binding events. Cathodic shifts registered for drug–DNA complex formation are typical for electrostatic interactions [47,48,49,50,51,52]. In the case of NPh-DOX-NGR, negative shifts of oxidation potentials of heterocyclic bases guanine, adenine, and thymine may be due to the influence of charged functional groups of the targeted peptide. The possible reason for the decrease in G, A, and T peak current signals might be due to the shielding of oxidizable groups of these bases, or a damage of these bases provoked by DOX itself (Figure 4A–C) [1,2,7,46].

Specific DNA–DOX (and DOX nanoderivatives) interactions produce registered changes of the electron transfer on the electrode interface and, as a result, the opportunity for assessing thermodynamic parameters, such as affinity constants and changes in Gibbs energy (ΔG) [53,54]. Electrochemical methods for DNA analysis and drug–DNA interaction possess high sensitivity and multiparametric assays for the calculation of thermodynamic parameters. The changes in the Gibbs free energy (ΔG) were calculated using the well-known equation ΔG = −RTln*K_b_* (Table 2).

Electroanalysis of DOX or DOX nanoderivative interactions with DNA revealed that no new electrochemical signals appeared after complex formation at constant DNA concentration and varying drug concentration (Figure 3A–C). The binding constants *K_b_* for the interaction processes between DNA and DOX nanoderivatives were calculated using the following equations for the reversible mode of interaction of DNA–drug (2, 3):

DNA + drug ⇆ [DNA*drug](1)

*K_b_* or summary (total) equilibrium constant can be deduced from this equilibrium as:

*K_b_* = [DNA*drug]/([DNA] × [drug]) (2)

or in logarithmic form:

(3) $$log\left(\frac{1}{\left[Drug\right]}\right)=logK_{b}+log\left(\frac{I\left(DNA*Drug\right)}{I\left(DNA\right)-I\left(DNA*Drug\right)}\right)$$

In electroanalytical methods, the concentration of DNA can be approximated by peak height calculated either for A, G, or T peaks. The concentrations of complexed drug [DNA*drug] were assumed to be proportional to the I(DNA*drug), and where I(DNA) and I(DNA*drug) were the peak currents of dsDNA in the absence and in the presence of DOX nanoderivatives, respectively [41,46,48], where the DNA concentration was constant for all experiments.

**Figure 5:**
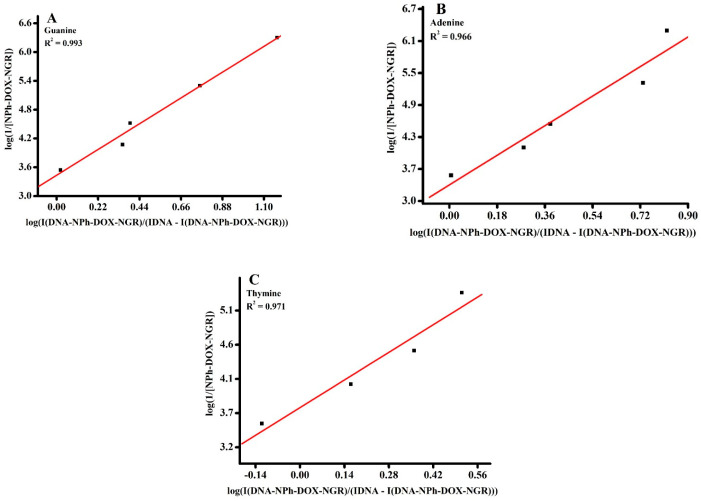
Relationship between log (1/[drug]) and log (1/[dsDNA*drug])/I[DNA] − I [dsDNA*drug] for NPh-DOX-NGR nanoderivative calculated for interaction based on (**A**) guanine; (**B**) adenine; and (**C**) thymine oxidation signals.

The double reciprocal plot of log (1/[dsDNA*drug]) vs. log (1/[drug]) is linear, and the association binding constant (*K_b_*) is calculated from the intercept on the vertical coordinate axis to the slope, as described in [11,12,13,34]. By this way, the binding constants of drug interaction with dsDNA were calculated for adenine, guanine, and thymine. The data are presented in Figure 5A–C, Figures S1 and S2, and are summarized in Table 2.

The *K_b_* values for doxorubicin are about 10^4^ M^−1^ and reflect the intercalation type of binding [12,13,46]. The interaction of G with DOX was not pronounced, so we were not able to calculate *K_b_* for this binding. Other forms of doxorubicin, such as phospholipid composition NPh-DOX and phospholipid composition with the peptide (NPh-DOX-NGR), are characterized by lower values of binding constants of the order of 10^3^ M^−1^, which indicate the manifestation of electrostatic interactions (Table 2).

The DNA-mediated electrochemical coefficient of the toxic effect for DOX, NPh-DOX, or NPh-DOX-NGR within the wide range of concentration 0.5–290 µM can be estimated as a value of the percentage of nucleobase peak height decrease (k, %) using Equation (4), in accordance with [39,40,41]:

*k* = (*ks*/*kb*) × 100% (4)

where *ks* is the maximum values of the currents of the oxidative signals of the nucleobase after interaction with the DOX (or DOX derivatives), and *kb* (before) is the maximum values of the currents of the oxidative signals of the nucleobase before the interaction.

A drug does not have a toxic effect if *k* is higher than 85%, a moderate toxic effect if the k parameter is between 50 and 85%, and a toxic effect if *k* is below 50% [39,40,41].

**Figure 6:**
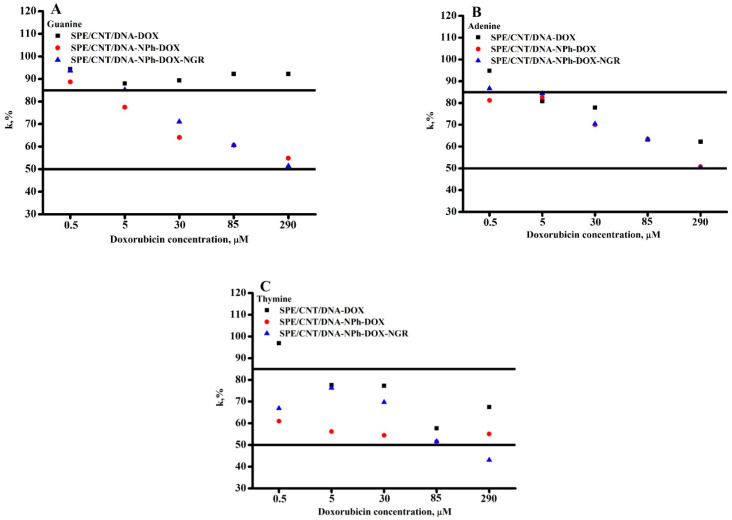
The influence of DOX in the concentration range of 0.5–290 µM on DNA oxidation signals of guanine (G), (**A**), adenine (A), (**B**) and thymine (T), (**C**), k, %—DNA-mediated electrochemical coefficient of toxic effect. Data represent average values of three independent experiments.

The electrochemical coefficient of the toxic effect of DOX, NPh-DOX, or NPh-DOX-NGR, based on the influence on oxidation peak current of DNA nucleobases, revealed nontoxic or moderate toxic action of DOX and DOX nanoderivatives in the 0.5–290 µM therapeutic window [52,55] (Figure 6).

## 3. Discussion

In this paper, a sensitive electrochemical method for the quantification of DOX with a limit of detection of 2.62 µM was developed. Based on the linear dependence of the intensity of electroreduction/electro-oxidation of doxorubicin on the square root of the scan rate, it was proven that the process is controlled by diffusion. DOX exhibited a single anodic peak at E = +0.260 ± 0.006 V. For the investigation of drug–DNA interaction, we used differential pulse voltammetry (DPV) technique and DNA sensor constructions. The modification of screen-printed electrodes with carbon nanotubes permits to separately register DOX and three heterocyclic bases of DNA molecules during the interaction processes. Electrochemical platforms represent a sensitive and informative approach for the investigation of DNA–drug interaction in electrode systems. Examining the electrochemical signals of DNA or DNA–drug complexes before and after binding establishes the interaction and helps in mechanism elucidation. A study of the effect of doxorubicin and doxorubicin nanocompositions on DNA confirms the intercalation mode of DOX on the DNA molecule. Such an approach helps to confirm the intercalative interaction mode for DOX by a shift in the oxidation peak towards the positive potential as a thermodynamically unfavorable process, as was described earlier [1,2,3,4,5,6]. DOX can shield heterocyclic bases, which leads to a potential shift to the anode region. The negative (cathodic) shift of the peak potentials for the phospholipid composition with the addressed peptide (NPh-DOX-NGR) is typical for electrostatic interactions due to the charged functional groups of the targeted peptide NGR. The calculated *K_b_* values also confirmed the binding type of interaction: for doxorubicin, it is about 10^4^ M^−1^ and reflects the intercalation type of binding. The phospholipid composition NPh-DOX and the phospholipid composition with the peptide (NPh-DOX-NGR) are characterized by a binding constant on the order of 10^3^ M^−1^, which indicates the manifestation of electrostatic interactions. The NGR peptide has affinity for aminopeptidase N (known as the CD13 marker on the membrane surface of tumor cells). It was shown that the new doxorubicin derivative with NGR peptide (NPh-DOX-NGR) is able to penetrate and internalize to a greater extent in HT-1080 (CD13-positive) cells [24]. NGR peptide did not reduce anticancer activity of DOX [56,57]. Doxorubicin embedded in nanoparticles containing NGR can enhance the antiproliferative effect of NGR/UT-L on HT-1080 cells [56]. The introduction of NGR peptide in the delivery system increases antitumor activity in vitro in comparison with free DOX, and significantly inhibited tumor growth in naked mice with xenotransplanted HT-1080 tumors [56]. In our comparative investigations, we studied the interaction of DOX, phospholipid composition NPh-DOX, and phospholipid composition with the peptide (NPh-DOX-NGR) with DNA as a model for pharmacogenomics. Hajian et al. investigated the electrochemical parameters of DOX and DOX in the presence of DNA using a multiwalled carbon nanotube-modified platinum electrode [57]. Cyclic voltammetry studies also showed an intercalation mechanism with a binding constant of 1.12 × 10^5^ M^−1^ [57]. There are many viewpoints on the mechanism of DOX binding with DNA molecules. Some results show that DOX intercalates into the base pair of cytosine and guanine of DNA, while other research data indicate that DOX intercalates into the base pair of adenine and thymine [6]. These conclusions depend on the method of investigation, such as fluorescence correlation spectroscopy, surface-enhanced Raman scattering, and UV-resonance Raman spectroscopy. Infrared spectroscopy added the electrostatic type of interaction with the phosphate group of DNA molecules [6]. DOX can bind with both base pairs of AT and GC by comparing the equilibrium constants calculated based on circular dichroism, viscometry, differential scanning calorimetry, fluorescence, isothermal titration calorimetry, and T-jump relaxation measurement; the fast step is the groove binding in the AT region, and the slow step is the intercalation into the GC region [1]. Based on our electrochemical results, we can conclude that DOX itself interacts predominantly with adenine and thymine. Phospholipid composition NPh-DOX and phospholipid composition with the peptide (NPh-DOX-NGR) interact with guanine, adenine, and thymine. It is very important to keep in mind that with the introduction of an addressed peptide in drug composition as additional function or gain of function can dramatically change the mechanism of drug interaction with the biological goal molecule.

## 4. Materials and Methods

The following reagents were used in this work: monosubstituted potassium phosphate (Reakhim, Moscow, Russia), sodium chloride (Reakhim, Moscow, Russia), single-walled carbon nanotubes 0.4 wt.%, stabilized with carboxymethylcellulose 0.6 wt.% (Tuball Batt H2O 0.4 wt.%, OCSIAL Ltd., Oksial Additives NSK LLC, Novosibirsk, Russia, https://ocsial.com); double-stranded fish sperm DNA (dsDNA) was bought from Sigma-Aldrich (D3159, Tokyo, Japan). The purity of the DNA stock solution was confirmed by taking the absorbance ratio of A260/A280, which was found to be in the range of 1.8–1.9, indicating there was no protein contamination in the DNA. The substance of doxorubicin with a 99% g/c purity was obtained in Omutninskaya Scientific Pilot-Industrial Base (JSC Omutninsk Scientific Experimental-Industrial Base, Vostochnyi, Russia). DOX embedded into phospholipid nanoparticles (NPh-DOX) and DOX embedded into phospholipid nanoparticles with peptide ligand Asn-Gly-Arg (NGR) attached to the surface of liposomes (NPh-DOX-NGR) were prepared as described earlier [22].

Electrochemical measurements were carried out using Autolab 302N potentiostat/galvanostat (Metrohm Autolab, Utrecht, The Netherlands) equipped with the NOVA software (version 2.0).

We used three-contact SPEs (Color Electronics, Moscow, Russia, http://www.colorel.ru) with working and auxiliary graphite electrodes, and a silver/silver chloride reference electrode. Working electrode diameter is 0.2 cm (area 0.0314 cm^2^). All potentials are given relative to a silver/silver chloride reference electrode (vs. Ag/AgCl). Analytes were prepared in 0.1 M potassium phosphate buffer (pH 7.4) containing 0.05 M NaCl; freshly prepared solutions were used in the electrochemical experiments.

The electrodes were modified with 2 μL of 1 mg/mL freshly prepared water dispersion of single-walled carbon nanotubes (SPE/CNT) Tuball Batt H2O 0.4 wt.% (preliminarily 6 times diluted in distilled water) and dried at room temperature for 25 min. The modified electrodes SPE/CNT were scanned in the potential range of 0.2–1.2 V (4 scans) in 0.1 M potassium phosphate buffer (pH 7.4) containing 0.05 M NaCl.

The experiments were performed under aerobic conditions at room temperature (25 ± 3 °C) and a horizontal regimen of measurement was used. To assess the reproducibility of the results for each concentration, at least 3 electrodes were used and the standard deviation was calculated. The standard deviation did not exceed ±10%.

For the investigation of DNA–drug interaction, a complex of DNA with DOX was formed at specified concentrations of pharmaceutical formulations and constant concentration of DNA at 1.5 mg/mL, and was incubated 10 min before adsorption onto the electrode surface [21,35,36]. Differential pulse voltammetry (DPV) was carried out in the potential range of 0.2–1.2 V, potential step 0.005 V, pulse amplitude 0.025 V, pulse duration 0.05 s, scanning frequency 0.05 s.

The DNA-mediated electrochemical coefficient of the toxic effect can be estimated at each DOX concentration as a value of the percentage of the G, A, or T peak height change (k, %) using Equation (4), as reported in [38,39,40,41]. To calculate the maximum peak current of the nucleobases’ electrochemical oxidation signals, a baseline correction was carried out using the NOVA software (version 2.0).

## Figures and Tables

**Table 1:** Parameters of DOX concentration dependences measured by DPV on the SPE/CNT.

E_Ox_, V	Concentration Range, µM	Slope, µA/µM	Equation for Linear Regression *	Limit of Detection, LOD, µM	Limit of Quantification, LOQ, µM	Correlation Coefficient, R^2^
0.260 ± 0.006	50 ÷ 317	0.011	I_0.260_ = 0.01207[DOX] + 0.01597	2.62	7.95	0.987

* I_0.260_ corresponds to the oxidative currents (peak heights) for DOX; [DOX] is a concentration of DOX in µM that was deposited onto the working surface area of SPE/CNT.

**Table 2:** Values of binding constant *K_b_* for DNA–drug complexes.

Drug, µM	Heterocyclic Base	*K_b_*, M^−1^	ΔG, kJ/mol
DOX 0.5–290	A	1.2 × 10^4^	−23.2
	T	1.6 × 10^4^	−24.0
NPh-DOX 0.5–290	G	1.0 × 10^4^	−22.7
	A	3.1 × 10^3^	−19.9
	T	5.4 × 10^3^	−21.2
NPh-DOX-NGR 0.5–290	G	3.8 × 10^3^	−20.4
	A	4.9 × 10^3^	−21.0
	T	4.0 × 10^3^	−20.5

## Data Availability

The data presented in this study are available on request.

## Supplementary Materials

The following supporting information can be downloaded at: https://www.mdpi.com/article/10.3390/molecules28145317/s1, Figure S1: Relationship between log (1/[drug]) and log (1/[dsDNA*drug])/I[DNA] − I [dsDNA*drug] for DOX calculated for interaction based on (**A**) adenine, (**B**) thymine oxidation signals; Figure S2: Relationship between log (1/[drug]) and log (1/[dsDNA*drug])/I[DNA] − I [dsDNA*drug] for NPh-DOX calculated for interaction based on (**A**) guanine, (**B**) adenine, (**C**) thymine oxidation signals.
