# Supplementary material for: Interaction of Doxorubicin Embedded into Phospholipid Nanoparticles and Targeted Peptide-Modified Phospholipid Nanoparticles with DNA
Source: Molecules. 2023 Jul 10;28(14):5317. doi: 10.3390/molecules28145317 (PMC10385298; doi:10.3390/molecules28145317)
Supplement: Supplementary file 1 [file molecules-28-05317-s001.zip › molecules-2441923-supplementary.pdf]

Interaction of Doxorubicin Embedded into Phospholipid Nanoparticles and Targeted Peptide-Modified Phospholipid Nanoparticles with DNA

[1] Institute of Biomedical Chemistry, Pogodinskaya Street, 10, Build 8, 119121 Moscow, Russia; veronicapunch@mail.ru (V.V.P.); kostryukova87@gmail.com (L.V.K.); tanya.bulko@mail.ru (T.V.B.)
[2] Faculty of Biochemistry, Pirogov Russian National Research Medical University, Ostrovitianov Street, 1, 117997 Moscow, Russia
* Correspondence: viktoria.shumyantseva@ibmc.msk.ru; Tel.: +7-499-246-5820

Supplement data

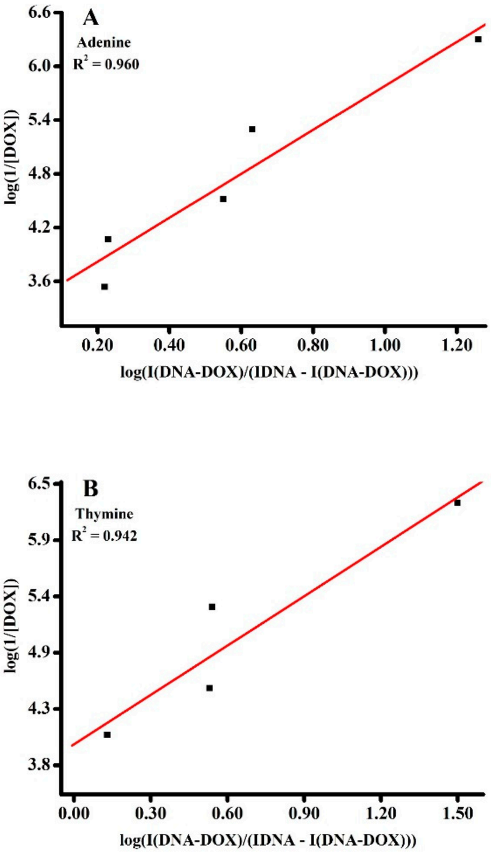

Figure S1. Relationship between log (1/ [drug]) and log (1/[dsDNA*drug])/I[DNA]-I [dsDNA*drug] for DOX calculated for interaction based on (A) adenine, (B) thymine oxidation signals.

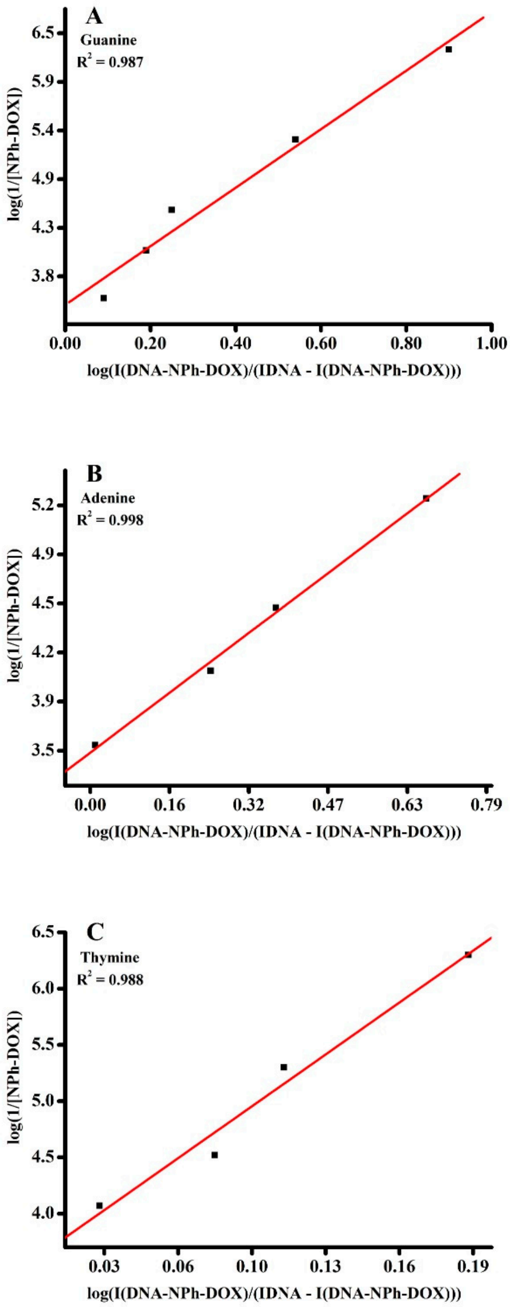

Figure S2. Relationship between log (1/ [drug]) and log (1/[dsDNA*drug])/I[DNA]-I [dsDNA*drug] for NPh-DOX calculated for interaction based on (A) guanine, (B) adenine, (C) thymine oxidation signals.
